# Comparison of milk production of dairy cows vaccinated with a live double deleted BVDV vaccine and non-vaccinated dairy cows cohabitating in commercial herds endemically infected with BVD virus

**DOI:** 10.1371/journal.pone.0240113

**Published:** 2020-10-01

**Authors:** Ellen Schmitt–van de Leemput, Lucy V. A. Metcalfe, George Caldow, Paul H. Walz, Christian Guidarini

**Affiliations:** 1 Vetformance, Clinique Vétérinaire, Mayenne, France; 2 Boehringer Ingelheim Vetmedica GmbH, Ingelheim, Germany; 3 Scottish Agricultural Colleges Veterinary Sciences Division St Boswells, Roxburghshire, Scotland, United Kingdom; 4 Department of Pathobiology, College of Veterinary Medicine, Auburn, Alabama, United States of America; University of Illinois, UNITED STATES

## Abstract

Daily milk production and reproductive performance of cows vaccinated with a live double-deleted Bovine Viral Diarrhoea Virus (BVDV) vaccine were compared to those of non-vaccinated cows, cohabitating in endemic BVDV herds. All animals in the treatment group were vaccinated on study day 0 irrespective of lactation or gestation status, while control animals did not receive any treatment. 1463 animals were enrolled in the study from four different farms in three different countries (UK, Italy, France). Endemic presence of BVDV in study herds was demonstrated by the detection of BVDV in the bulk tank milk, and seroconversion was evaluated at the beginning of the study. For individual animals, the day of calving was taken to be the start of lactation for the calculation of days in milk (DIM). The standard lactation period of 305 days was divided into three periods: early lactation (EL, from DIM 8 to DIM 102), mid lactation (ML, from DIM 103 to DIM 204 and late lactation (LL, from DIM 205 to DIM 305). For each farm and each lactation period, a mixed model statistical analysis was performed with daily milk production as response, and group, day as well as the interaction between those two factors as fixed factors. Chi-square test was used to compare abortion rate and prolonged inter-oestrous interval rate between treatment and control groups. A significant increase in milk production in the vaccinated group was observed in farms 1 (1.023 L/day) and 3 (0.611 L/day) during EL (p<0.001) and in farm 2 (1.799 L/day) during ML (P<0.001). In addition, at farm 2, vaccinated cows produced more milk than non-vaccinated cows starting from 80 DIM. No differences were found between groups in abortion rates or prolonged inter-oestrous interval rates. Data demonstrate that cows in herds endemically infected with BVDV and vaccinated with live double-deleted BVDV vaccine produce more milk; the difference in milk production occurs during early lactation.

## Introduction

Bovine Viral Diarrhoea Virus (BVDV) is a member of the genus *Pestivirus* within the family *Flaviviridae* [1]. The virus was first described in New York in 1946 and has been identified in most cattle-producing countries worldwide since [2, 3]. BVDV can be found in a wide range of body fluids, such as nasal discharge, urine, milk, semen, saliva, tears and foetal fluids [4]. The most important source of BVDV infection is persistently infected (PI) cattle [5]. PI animals are immunotolerant to the persisting virus and shed infectious virions throughout their life. In non-pregnant non-immune cattle, acute infections with BVDV result in transient viremia [6]. Infections of pregnant cattle provide the opportunity for the virus to cross the placenta causing foetal infection. The occurrence of foetal infection depends on the age of the foetus at the time of BVDV infection. Embryonic death, teratogenic effects in the foetus or the birth of a PI calf can be the result [7, 8]. PI calves can be small, weak and ill-thrifty but more often appear healthy and could go undetected within the herd [9].

Consequences of transient infection, in particular decreased fertility and immunosuppression, can be profound [10]. The effects of acute BVDV infection in female cows on reproductive performance have been considered to be the most costly and include reduced conception rate, embryonic death, abortion and congenital defects [8]. The underlying mechanisms of BVDV-induced infertility and reproductive disease are multifactorial, and can involve direct impacts of the virus on reproductive tissues, including the developing fetus, and indirect effects through immune system dysregulation [11]. BVDV or virus-specific antigens can be identified in reproductive tract tissues from infected cattle, and BVDV infection has been demonstrated to alter endocrine functions of reproductive tract tissues [11]. Indirectly, BVDV infection of naive cattle can render them more susceptible to secondary infections through immunosuppression [12]. BVDV is lymphotrophic, and transiently infected cattle can have immune system dysregulation due to the combined effects of immune cell depletion and diminished function of remaining immune cells [13]. Immune system cells of both the innate and adaptive immune responses can be affected during BVDV infection. Removal of BVDV-infected leukocytes (immunodepletion) by the immune system, destruction of immune cells by BVDV, and increased trafficking of immune cells into tissue sites of viral replication combine to result in leukopenia.

BVDV infection is an economically important disease of cattle; specific estimation of its costs are difficult [14]. Global economic reviews report losses which vary from £ 0–552 per cow per year (£ 2370 including outliers) [15]. Losses reported to be associated with BVDV include premature voluntary culling and reduced slaughter value, mortality losses, abortion and other reproductive losses, veterinary and treatment costs, the cost of additional labour and reduction in milk production [16]. The large variance in BVDV-related losses is not only dependent on characteristics relating to the infecting strain but also on both herd- and animal-level factors such as concurrent disease, management, and the immunity of the herd. Of major importance is the number of at-risk animals, at a critical phase of reproduction at the time of exposure to the virus. Reported costs vary substantially according to whether the infection is epidemic i.e. introduction of virus into seronegative, vulnerable cattle populations or endemic i.e. widespread throughout the herd [16, 17]. Severe financial loss has been reported in epidemic situations whereas, in herds with endemic BVDV, previous exposure of animals to the virus has resulted in the development of some level of natural immunity minimising the associated losses [16].

Reduced milk production is an important component of the economic losses due to BVDV [17]. The magnitude of the impact of BVDV upon milk production also depends on the nature of the infection. Reports of epidemic BVDV outbreaks describe sudden reductions in milk yield while a comparison of production levels at farms before and after BVDV eradication describe a general tendency for increase in milk production [18, 19]. Under endemic conditions, Moerman et al. found a significant reduction in milk yield experienced in cows seroconverting to BVDV compared with herd mates that did not seroconvert, and high antibody titres in bulk tank milk have been correlated with reduced milk production [20, 21]. BVDV has both direct effects on milk yield and indirect effects due to both increased returns to service causing prolonged calving intervals and higher disease incidence [17, 22]. Immune system dysregulation leading to increased clinical mastitis rate, feed energy being used for immune function, abortion and the combination of fever and decreased appetite have all been postulated to explain the impact on milk production [21].

The aim of this study was to evaluate whether vaccination against BVDV infection with a live, double-deleted BVDV vaccine (Bovela®) can prevent the reduction in milk yield in BVDV infected herds. For this purpose, a clinical trial was successfully conducted in commercial dairy farms. In the same herd, on the same day, half of the animals were vaccinated with Bovela® and the other half of the animals served as controls and received no treatment.

## Materials and methods

### Farm and animal selection

From January 2017 until December 2017, four commercial dairy farms with more than 100 lactating cows were included in the study in three different countries (farms 1 and 2: UK, farm 3: Italy and farm 4: France). The herds needed to be equipped with an individual daily milk production recording system (inline milk meters) and were required to have records of disease, treatment, insemination and animal movement data. Three farms used conventional milking systems (farms 1 (Metatron p21, GEA, Germany), 2 (Weighall, Dairymaster, Ireland) and 3 (AfiFlo 2000, Afimilk (Israel))) and one farm used automatic milking units (farm 4 (Ponderal, Lely, The Netherlands)). The dairy breed present on all farms was Holstein. Herds were excluded if they had been vaccinated with either an attenuated live BVDV vaccine (within 5 years prior to inclusion) or an inactivated BVDV vaccine (within 3 weeks before the start of the study). These exclusion criteria were selected based on the general consensus that the protection elicited by a live vaccine is broader and of longer duration [23]. Therefore, 3 weeks exclusion for an inactivated vaccine was to avoid potential interference with either milk production, which may be negatively impacted shortly after administration of an adjuvanted vaccine, or the immune response caused by two BVD vaccines being administered in close time proximity. A much longer exclusion of 5 years was chosen for live vaccines in order to prevent inclusion of herds that could still have broad protection induced by a previous live BVD vaccine.

Farm 3 reported a single use of a killed BVD vaccine on the cows in the lactating herd, which had been administered at seven months prior to SD 0. The other farms had not used any vaccine against BVDV prior to the start of the study. All female animals (lactating cows, dry cows and heifers) older than 10 months of age present at the farm on the day of vaccination were eligible for inclusion in the study. On the day prior to vaccination [study day minus 1 (SD-1)], a general health observation was performed and animals with an abnormal finding underwent clinical examination, including lameness evaluation. Unhealthy animals, including those with lameness grade 4 or 5 (severe lameness), were excluded from participation in the study; however, these animals remained present in the herd [24]. At SD0 471, 316, 499 and 177 animals were included in the study at farm 1, 2, 3 and 4, respectively. The total numbers of lactating cows, dry cows and heifers for the two treatment groups are described in Table 1, as well as the number of animals determined to be ineligible for enrolment. Average milk production was similar for farms 1, 2 and 4 (between 30 and 32 L/day/cow) and between 41 and 42 L/day/cow for farm 3.

**Table 1:** Distribution of animals to treatment groups according to their physiological status at the day of vaccination (SD0).

	All included animals			Lacting cows		Dry cows		Non lactating Heifers	
	Total (E SD-1)^a^	G1^b^	G2^c^	G1^b^	G2 ^c^	G1^b^	G2^c^	G1^b^	G2^c^
Farm 1	471 (2)	237	234	127	126	23	19	87	89
Farm 2	316 (11)	158	158	89	89	18	15	51	54
Farm 3	499 (0)	250	249	133	132	19	23	98	94
Farm 4	177 (4)	89	88	49	52	4	3	36	33

Some animals were excluded from participation to the study due to health reasons (E SD-1). Those animals did not integrate into either treatment group but remained present at the farm.

^a^Animals excluded at SD-1 (for health reasons) that remained present at the farm

^b^Treatment group 1: Vaccination with 2ml of Bovela®, intramuscular injection

^c^Treatment group 2: No treatment

Ultimately, to be eligible for inclusion, a proof of BVDV infection within the herd during the six months prior to the start of the study was required. Animals were housed and fed according to the usual standards for each farm. The duration of the study was 365 days, starting from the day of vaccination [Study Day 0 (SD0)], and the study was performed in compliance with good clinical practice (GCP) guidelines, which ensure the maintenance of acceptable levels of animal welfare. The commercial product was administered as part of standard disease prevention measures, in accordance with the summary of product characteristics guidelines, by registered veterinary practitioners. For these reasons, and the absence of invasive procedures, no further approval from animal research ethics committee was required.

### Proof of endemic BVDV infection

Herds were considered to be endemically infected with BVDV if the virus was detected by polymerase chain reaction (PCR) in the bulk tank milk within the six months preceding SD0. The samples were collected in tubes containing a preservative agent (bronopol) and shipped at ambient temperature to the laboratory within 24 hours. The presence of BVDV in milk and serum was detected by PCR using a commercial kit (VetMAX Gold BVDV Detection kit, Thermo Fisher Scientific, USA). According to the manufacturer, this pan-pestivirus assay is capable of detecting other pestiviruses such as border disease virus. This kit employed a one-step real time RT-PCR protocol. One-step real time reverse transcriptase-PCR allows reverse transcription followed by PCR amplification and fluorescent binding of genotype-specific probes in a single well. Samples were considered positive for a target if the cycle threshold (Ct) value was ≤ 37. Samples with Ct values > 37 were considered inconclusive and those with undetermined Ct values were considered negative.

At SD0, a screening blood sample was harvested from lactating cows (n = 10 per treatment group) on each farm in sterile dry sample tubes for detection of BVDV antibodies. Antibodies to BVDV in serum were detected by using an indirect enzyme linked immunoassay (IDEXX BVDV Total AB Test). Samples were taken to be positive for antibodies to BVDV when the sample optical density (OD) divided by the OD of the positive control minus the OD of the negative control was greater than or equal to 0.30. For animals with a negative result in the ELISA test, a BVDV PCR was performed to detect possible presence of the virus. During the study, the presence of BVDV was monitored by monthly BVDV PCR analyses of the bulk tank milk. Bulk tank milk sampling procedure and analyses were identical to those described for samples collected during the six months prior to SD0.

### Vaccination procedure

At SD0, all healthy animals were restrained for vaccination either in head lockers or in reproduction rails. Animals were allocated to treatment group (G1 Vaccinated, G2 No treatment) according to order of appearance in the retention area; first animal was vaccinated and then every other animal either non-vaccinated or vaccinated accordingly. The G1 animals were vaccinated with one dose of double gene-deleted monovalent BVDV vaccine (Bovela^®^, Boehringer Ingelheim Vetmedica GmbH, Germany) by intra-muscular route in the neck region, using sterile single use materials. The G2 animals did not receive any treatment, thus there was no injection procedure. A placebo was not administered due to the requirement that Bovela be used within the terms of its marketing authorisation and no unjustified medical procedures be performed on any study animals, as discussed earlier.

Bovela^®^ is a modified live vaccine intended for the active immunisation of cattle against BVDV type 1 and type 2, only healthy animals should be vaccinated. Farm and laboratory personnel were blinded to treatment groups. Investigators (local practising veterinarian) and study monitors were not blinded to allocation to treatment groups as the study parameters measured were objective.

### Observations of the animals during the study

The investigator was responsible for all aspects of the conduct of the study at the individual study site during the entire duration of the study (365 days). The farmers were encouraged to contact the investigator for health issues of the study animals at any time. At the farmer’s judgement, the animals were examined and treated by either the farmer or the investigator. All incidences of disease were reported to the investigator at least once a month and further investigations conducted when deemed necessary. The treatment data from the official farm register was used, either in a paper version (farm 2) or in an electronic version (farms 1. 3 and 4). *Post-mortem* examination of animals that died during the study period, including abortions and stillbirths were performed according to the accredited regional veterinary laboratories standard protocol. In all cases, the minimum requirement was that fetal samples and blood samples of the dam were analysed for the presence of BVDV antigens and antibodies. Other diseases such as Neospora, Q fever, Salmonellosis, Leptospirosis and Brucellosis were analysed according to the decision of the investigators and farmers.

### Data collection

Data on daily milk production, reproductive performance and animal movements (buy in, calving, dry off, culling) were collected for 365 days, starting on SD0. The daily milk yields per cow were measured by inline milk meters. For each farm, the equipment was tested and calibrated before the start of the study by a local accredited organisation. Reproductive performance was measured by the abortion rate (number of abortions during the study / number of lactating cows at SD0*100) and embryonic mortality rate. Since embryonic mortality is associated with increased oestrous cycle length, embryonic mortality rate was estimated from the number of cycles of more than 24 days, referred to as prolonged inter-oestrous intervals [25]. The interval length was defined by the number of days between either two inseminations or an oestrus observation (not followed by insemination) and insemination on the subsequent observed oestrus. Regular intervals were represented by cycle length of 18 to 24 days, prolonged inter-oestrous intervals by cycle lengths of 25 to 80 days. The prolonged inter-oestrous interval rate was calculated as the number of prolonged inter-oestrous intervals / (normal oestrous intervals + number of prolonged inter-oestrous intervals) * 100. Data were exported on a monthly basis (fortnightly for milk production data) from the herd management computer software or a national database (Insemination and animal movement, UK) to the investigator. For each lactation, the milk yields from 7 days after calving (DIM) until 305 DIM were considered for analyses. If animals experienced lactation lengths of less than 305 days, the day before dry-off was excluded from the data set. Lactation lengths of less than 30 days were excluded from analyses. Also, milk production data during periods of disease treatment and antibiotic withdrawal time were not analysed from the day before the start of the treatment until the day following the conclusion of the withdrawal time. Lactating cows that were vaccinated close to dry off experienced two lactations during the study period (one before dry off and one after dry off), both lactations were included in the analysis. Lactation rank refers to the lactation number that the cow is in at the time of daily milk production being recorded.

### Data analysis

For individual animals, the day of calving was taken to be the start of the lactation (DIM 1). The standard lactation period of 305 days was divided into three separate periods: early lactation (EL, from DIM 8 to DIM 102), mid lactation (ML, from DIM 103 to DIM 204 and late lactation (LL, from DIM 205 to DIM 305). For each farm and each lactation period, a mixed model was used to analyse daily milk production. Group and day, as well as the interaction between those two factors, were considered fixed factors in the model. Repeated measures were made on each cow across days, and days were modelled as a categorical variable. To capture the correlations between observations from the same subject, the autoregressive (AR(1)) variance and co-variance structure was used. The compound symmetry (CS) was used if the models suffered from a lack of convergence. The lactation rank was considered as a covariable in the model. If the interaction between group and day was significant, G1 and G2 cows were compared for each day. If the interaction between group and day was not significant, the interaction was removed from the model and G1 and G2 cows were compared with all days confounded. Normality of residuals was checked by visualization of the Pearson residuals distribution. For each farm (1, 2, 3 and 4) a Chi-square test was performed to compare abortion rate and the prolonged inter-oestrous interval rate between G1 and G2. If the theoretical numbers observed were inferior to 5, a Fisher Exact test was used. Statistical analyses were performed with a type I error set at α = 5%, with two-tailed tests, using SAS BASE 9.4 SAS/STAT 13.1 (SAS Institute Inc., Carry, NC, USA).

## Results

### Proof of endemic BVDV infection

BVDV was detected in the bulk tank milk by PCR from all farms on at least one occasion during the six months prior to vaccination. All blood samples (100%) at SD0 (n = 20 per farm) were positive for antibodies against BVDV in farms 2, 3 and 4. On farm 1, 75% (15/20) of the serum samples were positive for antibodies against BVDV. Seroconversion can either be induced by natural infection with BVDV or by vaccination against BVDV. Cows located at farm 3 had been vaccinated against BVDV prior to inclusion in the study; thus for this herd, the seroconversion does not provide additional information regarding the nature of exposure to BVDV. BVDV was not detected by PCR in any of the serum samples that were without detectable antibody. In farms 2, 3 and 4, BVDV continued to be identified in the bulk tank milk in the samples collected each month (Table 2). On farm 2, farm-specific reasons meant that results were not available from approximately half of the monthly bulk tank milk sampling occasions.

**Table 2:** The results of the monthly BVDV PCR analyses of bulk tank milk.

	Prior to SD0^a^	SD0^b^	M1^c^	M2^c^	M3^c^	M4^c^	M5^c^	M6^c^	M7^c^	M8^c^	M9^c^	M10^c^	M11^c^	M12^c^
Farm 1	+	-	-	-	-	-	-	-	-	-	-	-	-	-
Farm 2	+	SE^d^	-	SE^d^	-	+	+	SE^d^	SE^d^	+	+	SE^d^	SE^d^	+
Farm 3	+	-	+	-	-	SE^d^	-	-	-	-	-	-	-	SE^d^
Farm 4	+	+	+	-	-	-	-	+	+	-	-	-	-	-

Samples were considered positive for BVDV if the cycle threshold (Ct) value is ≤37. Samples with Ct values >37 were considered inconclusive. Samples with undetermined Ct values were considered negative.

^a^Sample of the bulk tank milk taken at the most 6 months prior to the start of the study (SD0)

^b^Sample of the bulk tank milk taken on the day of vaccination

^c^Sample of the bulk tank milk taken during the first, second, third etc month after vaccination

^d^Result not available due to sample and shipment failures, sample error (SE)

### Vaccination

A total of 1463 animals were enrolled from the 4 farms in the study, and consisted of 734 G1 animals and 729 G2 animals (Table 1) distributed across the defined lactation stages (Table 2). Adverse events potentially related to the vaccination procedure were closely monitored. At 42 days after vaccination, one animal from G1 had an elevated rectal temperature (41°C) and purulent inflammation at the injection site. No pregnancy loss was reported during the 15 days after treatment in either G1 or G2. No adverse health event that occurred during the study was linked to the use of the Bovela® vaccine, according to the judgement of the investigators.

### Analyses of milk production data

**Fig 1:**
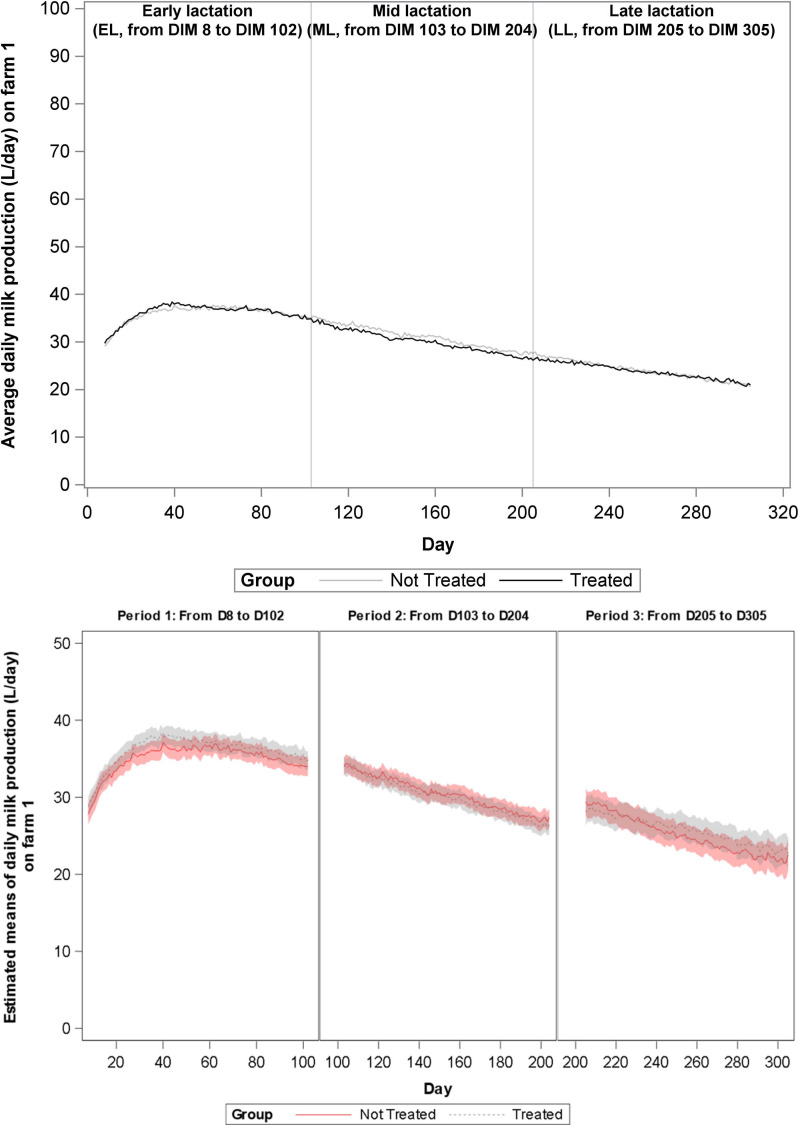
**A.** Evolution over time of average daily milk production (L/day) on Farm 1 for each treatment group. Analyses was performed per lactation period. During the first lactation period (early lactation, EL, 8–102 DIM) the milk yield of the vaccinated cows?animals was significantly different from the milk yield of the non-vaccinated animals (+1, 023 L/day/cow). During the second (mid-lactation, ML, 103–204 DIM) and the third lactation period (late lactation, LL, 205–305 DIM) no differences in milk yield were observed. **B.** Evolution over time of mean estimated daily milk production (L/day) on Farm 1 for each treatment group.

**Fig 2:**
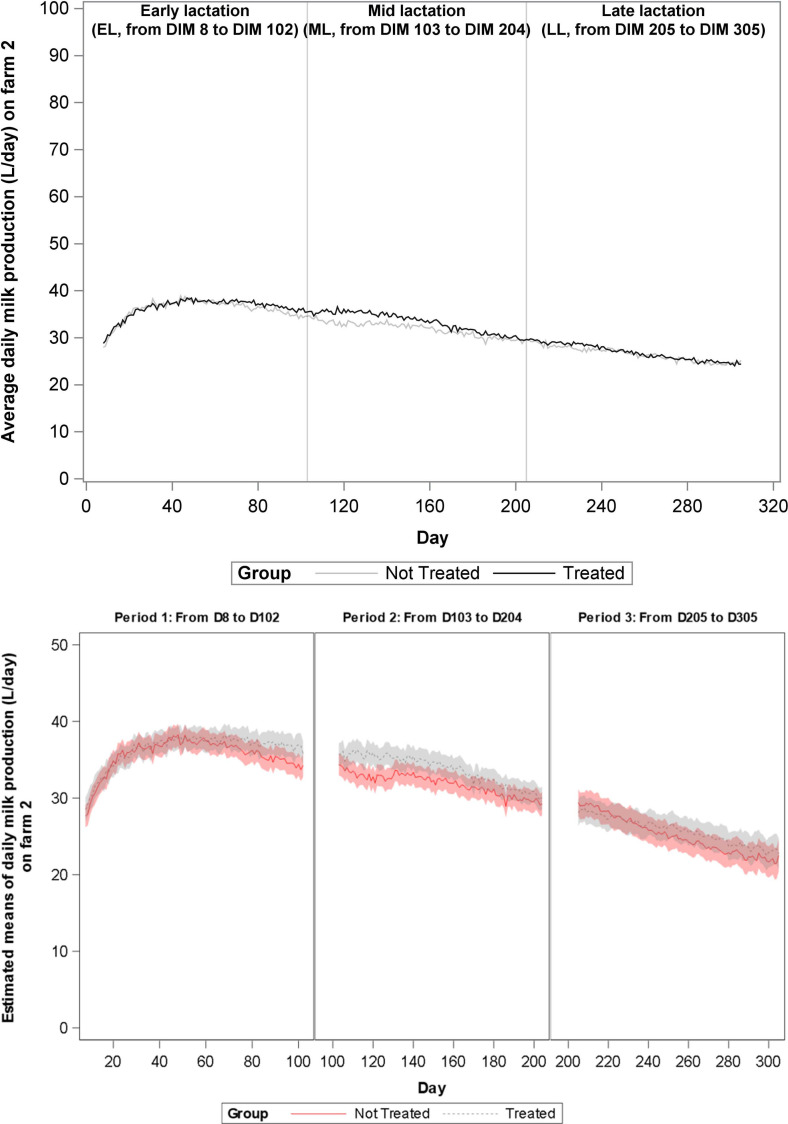
**A.** Evolution over time of average daily milk production (L/day) on Farm 2 for each treatment group. Analyses was performed per lactation period. During the second lactation period (mid-lactation, ML, 103–204 DIM) the milk yield of the vaccinated animals was significantly different from the milk yield of the non-vaccinated animals (+1,8 L/day/cow). During the first (early lactation, EL, 8–102 DIM) and the third lactation period (late lactation, LL, 205–305 DIM) no differences in milk yield were observed. **B.** Evolution over time of mean estimated daily milk production (L/day) on Farm 2 for each treatment group.

**Fig 3:**
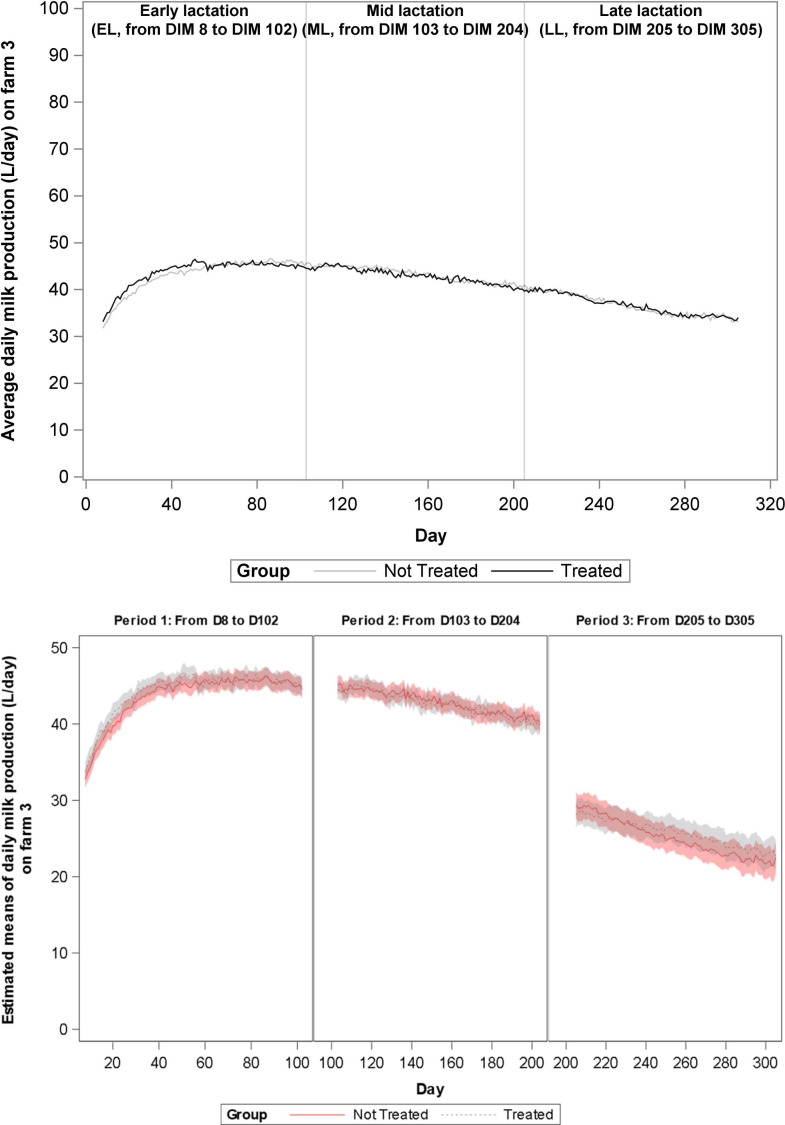
**A.** Evolution over time of average daily milk production (L/day) on Farm 3 for each treatment group. Analyses was performed per lactation period. During the first lactation period (early lactation, EL, 8–102 DIM) the milk yield of the vaccinated animals was significantly different from the milk yield of the non-vaccinated animals (+0,6 L/day/cow). During the second (mid-lactation, ML, 103–204 DIM) and the third lactation period (late lactation, LL, 205–305 DIM) no differences in milk yield were observed. **B.** Evolution over time of mean estimated daily milk production (L/day) on Farm 3 for each treatment group.

**Fig 4:**
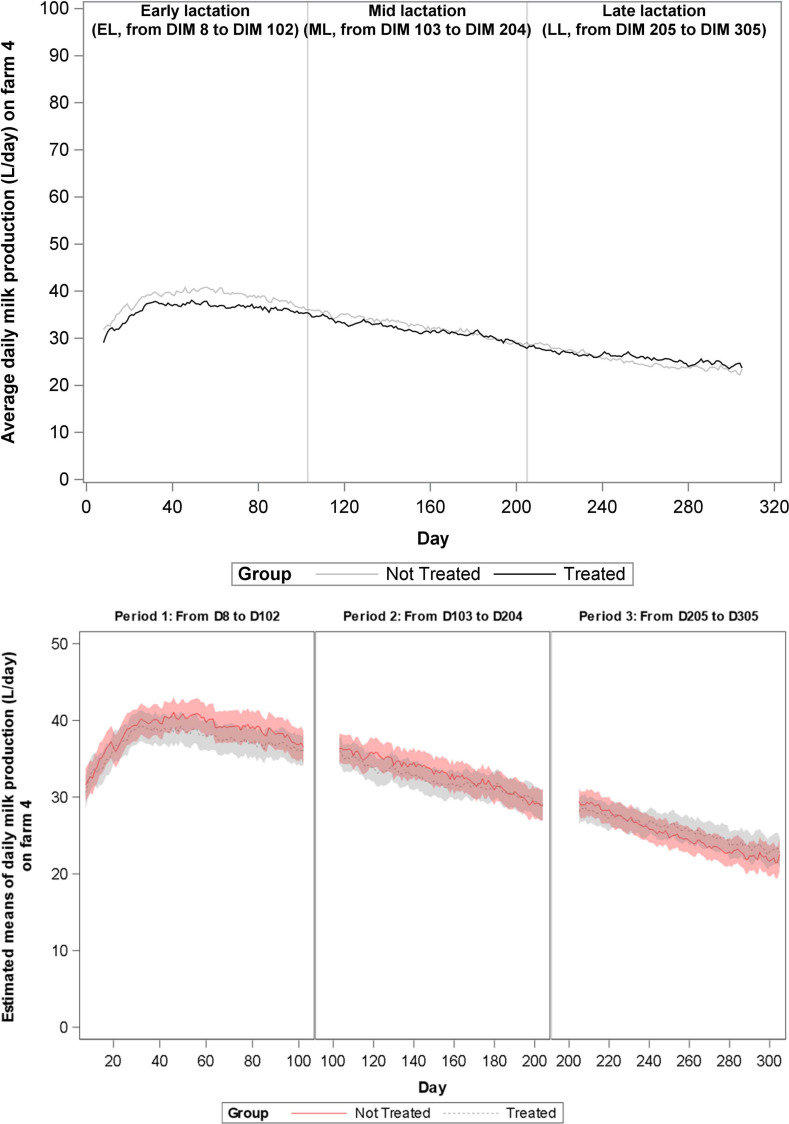
**A.** Evolution over time of average daily milk production (L/day) on Farm 4 for each treatment group. Analyses was performed per lactation period. During the first lactation period (early lactation, EL, 8–102 DIM), the second (mid-lactation, ML, 103–204 DIM) and the third lactation period (late lactation, LL, 205–305 DIM) no differences in milk yield were observed. **B.** Evolution over time of mean estimated daily milk production (L/day) on Farm 4 for each treatment group.

During the total study period of 365 days, milk production data were eligible for analyses from 1197 cows, and these cows represented 1559 lactations. Data from 28 lactations (1,9%) could not be used due to short lactation length. For these cows, either vaccination occurred less than 30 days before dry off or the animal calved close to the end of the study, such that less than 30 days of milk production data was collected. Distribution of the lactations according to treatment group, lactation rank and lactation stage are presented in Table 3. The change over time of average daily milk production on farms 1–4 is shown in Figs 1A–4A, and of mean estimates of daily milk production, after adjustment for lactation rank, in Figs 1B–4B, respectively. A significant increase in milk production was observed in G1 cows as compared to G2 cows in farms 1 (1,023 L/day, P value = 0.001) and 3 (0,611 L/day, P value = 0.011) during EL and in farm 2 (1,799 L/day, P value<0.001) during ML. At farm 2, G1 cows produced more milk than the G2 cows starting from 80 DIM. No other significant differences in milk production were found.

**Table 3:** Distribution of lactations eligible for analyses (n = 1599, produced by 1197 animals) according to treatment group, lactation rank and lactation stage.

Lactation period	Lactation rank	Farm 1			Farm 2			Farm 3			Farm 4		
		G1^b^	G2^c^	Total	G1^b^	G2^c^	Total	G1^b^	G2^c^	Total	G1^b^	G2^c^	Total
**EL**^a^	**1**	56	42	**98**	46	42	**88**	83	92	**175**	31	24	**55**
	**2**	35	54	**89**	37	45	**82**	48	51	**99**	14	16	**30**
	**3 and more**	93	78	**171**	34	41	**75**	73	79	**152**	27	33	**60**
	**Total**	**184**	**174**	**358**	**117**	**128**	**245**	**204**	**222**	**426**	**72**	**73**	**145**
**ML**^a^	**1**	61	47	**108**	51	42	**93**	65	67	**132**	29	24	**53**
	**2**	36	52	**88**	30	44	**74**	56	58	**114**	14	11	**25**
	**3 and more**	78	70	**148**	34	45	**79**	71	80	**151**	28	32	**60**
	**Total**	**175**	**169**	**344**	**115**	**131**	**246**	**192**	**205**	**397**	**71**	**67**	**138**
**LL**^a^	**1**	52	39	**91**	46	39	**85**	53	58	**111**	31	24	**55**
	**2**	36	42	**78**	29	37	**66**	55	51	**106**	18	18	**36**
	**3 and more**	63	54	**117**	29	29	**58**	62	73	**135**	27	27	**54**
	**Total**	**151**	**135**	**286**	**104**	**105**	**209**	**170**	**182**	**352**	**76**	**69**	**145**

^a^Early lactation: 8 to 102 DIM, ML: Mid lactation: 103–205 DIM, LL: Late lactation: 206–305 DIM

^b^Treatment group 1: Vaccination with 2ml of Bovela®, intramuscular injection

^c^Treatment group 2: No treatment

### Analyses of reproductive performance

Abortion rates and prolonged inter-oestrous interval rates at farms 1 to 4 varied between 2.0 and 6.4% and 45.7 and 73.0%, respectively. No differences were found between groups. *Leptospira hardjo*, *Neospora caninum* and Bovine Herpesvirus-1 were the pathogens most commonly suspected as being the causes of abortion based on the laboratory reports. In no cases were BVDV antigen detected in an abortion sample.

## Discussion

The aim of this study was to measure the effect of vaccination with a novel commercially-available double gene-deleted monovalent BVDV vaccine (Bovela®) on milk production in commercial dairy herds with evidence of exposure of the milking herd to BVDV. Since milk production is importantly influenced by farm-related factors such as nutrition, housing and management, we sought collaboration with farms in which large numbers (>100 lactating dairy cows) of vaccinated animals and control animals could cohabitate under identical farming conditions [26]. Numerous farms in different European countries were screened for BVDV infection, and while many farms were positive for BVDV, only very few were either eligible or opted for inclusion. In Europe, awareness of the risks of BVDV is high with most European countries actively encouraging farmers to participate in control and prevention strategies [27, 28]. Many potential candidate farms already used a live vaccine against BVD which prevented their inclusion in the study. Furthermore, once BVDV infection within a herd was confirmed, the investigator visited the farm owner to explain the study protocol. The investigators’ objectives were to explain the disease, the options for control, and finally the possibility of participating in the study. A proportion of the farm owners opted for immediate vaccination of the herd; this decision was for obvious ethical reasons supported by the investigator. Unfortunately, these factors contributed to preventing the study from reaching over 3000 animals per group, the target number.

In order to investigate the efficacy of Bovela® in preventing milk production losses in commercial herds with endemic BVDV, the first necessity was to establish the presence or absence of BVDV infection throughout the duration of the study. Lactating cow serological screening reported that 75–100% of samples were positive for antibodies against BVDV, indicating that the herds contained a PI animal [29]. In addition, at 3 of the 4 farms, positive bulk tank milk PCR samples provided evidence of the presence of at least one PI animal existing within the lactating herd, confirming the continuing presence of BVDV. Once PIs are detected in the milking herd, many animals will have been exposed and developed some level of natural immunity. The serological screening results supported this assumption and suggested that the study herds had been infected with BVDV for a considerable amount of time. Therefore, it is important to consider the impact of herd immunity on the study results. While 100% herd immunity is required to control BVDV, there will undoubtedly be an impact on BVD epidemiology of vaccinating half a herd [30]. Vaccination indirectly protects unvaccinated animals by reducing infection transmission opportunities [31]. Following would be the assumption that the number of susceptible animals in the non-vaccinated group was likely to have been so low, in addition to this reduced transmission potential, such that no effect on milk production of vaccination against BVDV would be observed. Interestingly, in none of the farms were all bulk tank milk samples positive during the entire study period and BVDV was not detected in the bulk tank milk (BTM) PCR samples from farm 1 subsequent to the initial positive sample taken prior to study day 0. A negative BVDV PCR result does not necessarily equate to absence of BVDV infection in the herd, just that the infected individual was not contributing to the BTM at that time point [29]. PI animals may leave the milking herd temporarily when dried off or permanently if culled; milk may be withheld from PI animals that are ill or under-going treatment. New PI animals can also be added to the herd from a new age cohort or through purchase. Furthermore, there is no published information on the detection rate of transient BVDV infection by PCR analyses of the BTM. Studies comparing individual animal BVDV shedding, measured by individual milk or blood samples, and the presence of BVDV in the BTM may provide information to improve the detection of BVDV in dairy herds. Follow-up testing aimed at eradicating BVDV from farm 1, after completion of the study period, confirmed the presence of BVDV throughout the duration of the study. On this farm, antigen ELISA testing of ear notch samples indicated that four of 212 youngstock tested were positive on initial test and had a repeat positive or had died upon retest 3 weeks later. These animals were born prior to the start of the study or during the early study phase.

The study was designed to evaluate the efficacy of Bovela® in protecting dairy animals from the negative impact of BVDV on milk production, during the 12 months following vaccination. With the objective to mimic field conditions, all animals (lactating cows, dry cows and heifers) of the treatment group (G1), were vaccinated on the same day (SD0), regardless of their age or reproduction status. No drop in milk production in the period immediately after vaccination was observed when compared to the control animals. An important finding when considering that the control group did not receive any placebo i.e. did not undergo an injection procedure.

Cows vaccinated with Bovela® produced more milk than the non-vaccinated cows on farms 1, 2 and 3 during the first 100 days of lactation, significantly more during early lactation on farms 1 and 3, and from 80 days until 200 days on farm 2. No improvement in milk production after vaccination with Bovela® was found in farm 4 (Fig 4). Farm 4 used an automatic milking system (robotic). Data based on faecal glucocorticoid metabolite excretion suggests that automatic milking is less stressful than conventional milking [32]. It may be that cows exposed to less stressful management conditions are better able to cope with the impact of BVD on immune function during early lactation.

Although the reduction of milk production due to both epidemic and endemic BVDV has been reported before, the observation that vaccinated animals produce more milk than cohabitating non-vaccinated herd mates in endemic BVDV herds is novel [18, 19]. Furthermore, the demonstration of a significant difference in milk production between vaccinated and non-vaccinated animals despite the expected low number of susceptible animals in the non-vaccinated group, and the failure to reach the target inclusion number is important. Indirect milk losses in BVDV infected herds have been speculated to be due to increased disease incidence due to immune system dysregulation and increased abortion rates [21]. However, abortion rates were not different between vaccinated and non-vaccinated animals in this study, and no difference was found in incidence of disease, which was not surprising as in no cases was BVDV infection suspected as being the cause of abortion and all samples were negative for BVDV antigen (data not shown). Interestingly, the differences in milk production in our study were observed during early lactation.

Due to the study design, early lactation does not coincide with the first period after vaccination. On SD0, animals at different stages of the lactation cycle entered the study at the same time; lactating animals, dry cows, pregnant heifers and heifers that would experience future pregnancies. Accordingly, milk production data were not analysed relative to the start of the study but relative to the DIM of individual cows according to the three stages of lactation: EL, ML and LL. Therefore, resulting changes to the epidemiology of BVDV infection due to vaccination of 50% of the herd are not likely to have been responsible for the observed difference in milk production.

Early lactation is a critical phase of the lactation cycle. Modern dairy cows experience an energy deficit during early lactation and this period is associated with impaired immune function. Recently, Contreras et al. suggested that inadequate adipose tissue remodelling that accompanies transition and early lactation compromises immune function [33]. Potentially, the observed difference in milk production between the vaccinated and non-vaccinated animals that cohabitate in the same herd is due to the direct effect of BVDV on energy uptake and utilisation in the non-vaccinated group. Infection with BVDV causes immunosuppression; stimulation of immune function and antibody production are highly energy-consuming. The authors believe that the increased milk production in the cows vaccinated with Bovela® may be due to the prevention of the immunosuppressive effects of BVDV, i.e.

- the additional stress on the immune system caused by BVDV added to the already impaired immune function during early lactation competes with bodily resources otherwise used for milk production
- secondary infections encountered during that period which have a negative impact on feed intake and production.

Data from this study demonstrates that, even under the condition of endemic BVDV infection, in three out of four commercial herds with cohabitating Bovela®-vaccinated and non-vaccinated animals, the vaccinated cows overproduced the non-vaccinated cows by 0,61 to 1,8 l/day during early lactation. These findings, for periods of respectively 94 to 101 days (EL, 8–102 DIM and ML 103–204 DIM), give a compounded amount of gain in milk produced of 57,43 L to 181,69 L. It is difficult to precisely quantify the financial gain associated with this increased production due to the variation of milk price in different countries, but if we take into consideration average milk price in the European Economic Area (EEA) during the first quarter 2020, the financial gain would range from 20 to 63 € per cow per lactation [34]. Since the study was performed with a single vaccine, it is unknown how other live vaccines would perform under the study conditions.

The findings of this study are additional considerations for the cost-benefit analyses of BVDV control programs. In addition, the observations provide an additional motivation for farmers to adhere to BVDV control programs. Especially when this finding is combined with the observation that in none of the herds were differences found in important reproductive parameters and incidence of disease, i.e. farm data did not demonstrate the problems that usually signify BVDV infection. Thus, BVDV infection can negatively impact milk yield without affecting the reproductive performance of a herd; milk production losses may be hidden.

In summary, cows in herds endemically infected with BVDV and vaccinated with Bovela® produce more milk than the non-vaccinated cows in the same herds. The difference in milk production occurs during early lactation.

## Supporting information

S1 Dataset Farm 1 full data set audited. (XLSX)

S2 Dataset Farm 2 full data set audited. (XLSX)

S3 Dataset Farm 3 full data set audited. (XLSX)

S4 Dataset Farm 4 full data set audited. (XLSX)

S1 File (DOCX)

S2 File BVD BTM PCR CT values FINAL. (XLSX)
