# Supplementary material for: Comparison of milk production of dairy cows vaccinated with a live double deleted BVDV vaccine and non-vaccinated dairy cows cohabitating in commercial herds endemically infected with BVD virus
Source: PLoS One. 2020 Oct 1;15(10):e0240113. doi: 10.1371/journal.pone.0240113 (PMC7529212; doi:10.1371/journal.pone.0240113)
Supplement: S1 File — (DOCX) [file pone.0240113.s005.docx]

1) For each farm and each period: ANOVA table and Pearson residuals

Farm B period 1

| **Effect** | **P-value** |
| --- | --- |
| Group | 0.001 |
| Day | <0.001 |
| Lactation rank | <0.001 |

Farm B period 2

| **Effect** | **P-value** |
| --- | --- |
| Group | 0.259 |
| Day | <0.001 |
| Lactation rank | <0.001 |

Farm B period 3

| **Effect** | **P-value** |
| --- | --- |
| Group | 0.641 |
| Day | <0.001 |
| Lactation rank | <0.001 |

Farm C period 1

| **Effect** | **P-value** |
| --- | --- |
| Group | 0.039 |
| Day | <0.001 |
| Group*Day | 0.038 |
| Lactation rank | <0.001 |

Farm C period 2

| **Effect** | **P-value** |
| --- | --- |
| Group | <0.001 |
| Day | <0.001 |
| Lactation rank | <0.001 |

Farm C period 3

| **Effect** | **P-value** |
| --- | --- |
| Group | 0.207 |
| Day | <0.001 |
| Group*Day | 0.012 |
| Lactation rank | 0.032 |

Farm D period 1

| **Effect** | **P-value** |
| --- | --- |
| Group | 0.011 |
| Day | <0.001 |
| Lactation rank | <0.001 |

Farm D period 2

| **Effect** | **P-value** |
| --- | --- |
| Group | 0.400 |
| Day | <0.001 |
| Lactation rank | <0.001 |

Farm D period 3

| **Effect** | **P-value** |
| --- | --- |
| Group | 0.858 |
| Day | <0.001 |
| Lactation rank | 0.016 |

Farm E period 1

| **Effect** | **P-value** |
| --- | --- |
| Group | 0.095 |
| Day | <0.001 |
| Lactation rank | <0.001 |
| Group*Day | 0.022 |

Farm E period 2

| **Effect** | **P-value** |
| --- | --- |
| Group | 0.312 |
| Day | 0.003 |
| Lactation rank | <0.001 |

Farm E period 3

Note: The lactation rank was removed from the model (p-value on the model without interaction=0.392)

| **Effect** | **P-value** |
| --- | --- |
| Group | 0.653 |
| Day | 0.003 |
